# Epitope Mapping and In Silico Characterization of Interactions between Der p 7 Allergen and MoAb WH9

**DOI:** 10.1371/journal.pone.0071269

**Published:** 2013-08-05

**Authors:** Hsiao-Yun Tai, Jia-Kai Zhou, Hong Chou, Ming F. Tam, Yu-Sen Chen, Sheh-Yi Sheu, Horng-Der Shen

**Affiliations:** 1 Department of Medical Research and Education, Taipei Veterans General Hospital, National Yang-Ming University, Taipei, Taiwan, Republic of China; 2 Department of Life Sciences and Institute of Genome Sciences, National Yang-Ming University, Taipei, Taiwan, Republic of China; 3 Department of Biological Sciences, Carnegie Mellon University, Pittsburgh, Pennsylvania, United States of America; National Institute for Medical Research, Medical Research Council, London, United Kingdom

## Abstract

Der p 7 is an important house dust mite allergen. However, antigenic determinants of Der p 7 are largely unknown. The purpose of this study is to analyze the determinants of Der p 7 and determine the structural basis of interactions between Der p 7 and WH9, an IgE-binding inhibition mouse monoclonal antibody (MoAb). IgE and WH9-reactive determinant(s) was identified by immunoblot using allergen mutants. A 3-D binary complex structure of Der p 7 and WH9 was simulated with homology modeling and docking methods. Our results obtained showed that among the five Der p 7 mutants (S156A, I157A, L158A, D159A, P160A), serum no. 1045 with IgE-binding against Der p 7 exhibited a reduced IgE immunoblot reactivity against Der p 7 L158A and D159A mutants. WH9 showed reduced immunoblot reactivity against S156A, L158A, D159A and P160A and the observation was confirmed by immunoblot inhibition. The WH9-binding determinant on Der p 7 containing S156, L158, D159 and P160 assumes a loop-like structure. The structural model of the Der p 7-WH9 complex suggests residues S156, I157, L158, D159 and P160 of Der p 7 contribute to WH9 binding via potential hydrogen bonds, electrostatic and hydrophobic interactions. In conclusion, MoAb WH9 interacts with critical residues L158 and D159 of Der p 7 and inhibits IgE-binding to Der p 7. Results obtained advance our understanding on molecular and structural bases of the antigenicity of Der p 7, its interactions with MoAb WH9 and facilitate the design of safer immunotherapy of human atopic disorders.

## Introduction

There is a worldwide increase in the prevalence of human atopic disorders. Allergens cross-link mast cell-bound IgE antibodies can trigger a cascade of inflammatory and hypersensitive reactions. Characterization of IgE-binding determinants on allergens and delineation of the interaction modes between allergens and their specific antibodies at molecular and structural levels will enhance our understanding in disease mechanisms and development of effective therapeutic strategies towards these annoying human diseases.

We have identified and characterized the important group 7 allergens including Der p 7 and Der f 7 which share 86% amino acid sequence identity and induce IgE antibodies in about 50% of mite-sensitized asthmatic patients [1]–[5]. These two allergens are structurally similar to a human bactericidal permeability increasing protein (BPI)/lipopolysaccharide-binding protein (LBP) [6]–[8]. Their potential interactions with Toll-like receptors (TLRs) after binding lipopolysaccharide and other bacterially derived lipid ligands may contribute to their allergenicity.

Results from x-ray diffraction analysis of crystals containing allergen-IgE complexes can provide interacting details between allergens and IgE molecules. However, human IgE antibodies are polyclonal, their serum levels are low and their amino acid sequences are difficult to obtain. Therefore, even though the crystallographic structures of more than 50 allergens have been elucidated [9], only two models of allergen-human IgE-derived Fab fragments complexes are now available [9].

Recently, we determined the IgE-binding determinant(s) of Der f 7. We demonstrated that Asp 159 is a critical core residue for IgE-binding and contributes to IgE-mediated cross-reactivity between Der f 7 and Der p 7 [10]. We have previously prepared a series of mouse monoclonal antibodies (MoAbs) against group 7 mite allergens [2]–[5]. MoAb WH9 was raised against Der p 7 but binds also Der f 7 [2], [3]. This MoAb has been shown to inhibit, up to 60%, IgE-binding to Der p 7 [4]. The result suggests that the determinant(s) for WH9 and human IgE antibodies on Der p 7 may overlap. The amino acid sequences of the variable regions of MoAb WH9 can be inferred from the mRNA sequences encoding the antibody in hybridoma cells and used in structural modeling. The resulting model may mimic the paratope of an IgE that binds to a similar determinant on Der p 7.

In this study, we determined experimentally the Der p 7 antigenic determinants recognized by human IgE and MoAb WH9. We sequenced the variable regions of the heavy (VH) and light (VL) chains of WH9 and generated a structural model for the variable regions of this MoAb by homology modeling. Finally, we undertook molecular docking [11] to create a Der p 7-WH9 binary complex structure which provides insights into interactions between Der p 7 and its specific antibodies at molecular and structural levels. Our approach demonstrated in this study also provides strategies in developing immunotherapy against human atopic disorders.

## Materials and Methods

### Patients' sera

Sera (nos. 1045 and 1077) containing IgE antibodies against the group 7 dust mite allergens were collected from patients with a clinical history of bronchial asthma and stored in aliquots at −70°C until use [10]. Serum no. 862 from an asthmatic patient determined previously without IgE antibody against Der p 7 was also included as a negative control in immunoblotting. This study has been approved by the Institutional Review Board of Taipei Veterans General Hospital.

### Culture of hybridoma WH9

Hybridoma WH9 [2] was thawed from cryo-preserved stocks and cultured in RPMI1640 (Life Technologies GIBCO BRL, Grand Island, NY, USA) supplemented with 10% heat-inactivated fetal calf serum (FCS, Hyclone, Logan, Utan, USA) and antibiotics (penicillin 100 U/ml, streptomycin 100 µg/ml, GIBCO). Temperature was maintained at 37°C in 5% CO_2_ atmosphere. Culture supernatants containing MoAb WH9 (IgG2b, κ) were collected and stored in aliquots at −20°C. Antibody activity of the culture supernatants against the group 7 mite allergens was analyzed by immunoblotting. Culture supernatants containing MoAb HD19 against group 7 mite allergens [4] and MoAb FUM20 against fungal serine protease allergens [12] were prepared essentially as described and used as controls.

### Site-directed mutagenesis

Der p 7 mutants carrying single alanine substitute at S156, I157, L158, D159 or P160 were prepared essentially as described [10]. A plasmid encoding Der p 7 [1] (GenBank accession no. U37044) was used as template in polymerase chain reaction (PCR) mutagenesis experiments with primers listed in Table 1. The PCR products were purified and inserted into the pQE80 expression vector (Qiagen Inc., Valencia, CA, USA) and transformed into *E. coli* JM109 for recombinant proteins expression. The mutations on the plasmids were confirmed by DNA sequencing and the recombinant proteins were affinity-purified with Ni-NTA resin columns (Qiagen) according to the manufacturer's instructions.

**Table 1:** Primers used in PCR for preparation of Der p 7 mutants and for amplification of the variable regions of WH9 cDNA.

Name	Nucleotide sequence in 5′ to 3′-end orientation
Dp7S156A_forward_	5′-^451^ catattggtggtcttgcaattttggatcc ^479^-3′
Df7S156A_reverse_	5′-^479^ ggatccaaaattgcaagaccaccaatatg ^451^ -3′
Dp7I157A_forward_	5′-^456^ ggtggtctttcagccttggatccaattttcg ^487^-3′
Dp7I157A_reverse_	5′-^487^ cgaaaattggatccaaggctgaaagaccacc ^456^-3′
Dp7L158A_forward_	5′-^459^ ggtctttcaattgcggatccaattttcgc ^487^-3′
Dp7L158A_reverse_	5′-^487^ gcgaaaattggatccgcaattgaaagacc ^459^-3′
Dp7D159A_forward_	5′-^459^ ggtctttcaattttggccccaattttcgct ^488^-3′
Dp7D159A_reverse_	5′-^488^ agcgaaaattggggccaaaattgaaagacc ^459^-3′
Dp7P160A_forward_	5′-^466^ caattttggatgcaattttcgctgtc ^491^-3′
Dp7P160A_reverse_	5′-^491^ gacagcgaaaattgcatccaaaattg ^466^-3′
Heavy chain MoVH3	5′- cag gtc caa ctc gag cag (c/t)ct ggg (g/t)ct -3′
Heavy chain MoIgG2b	5′- ctc ctt act agt agg aca ggg gtt gat tgt -3′
Light chain Vκ4	5′- caa att gtt ctc acc cag tct cca -3′
Light chain kappa constant	5′- gat gga tac agt tgg tgc -3′

### Sodium dodecyl sulfate (SDS)-polyacrylamide gel electrophoresis and immunoblotting

Reactivities of purified recombinant proteins against human IgE and mouse MoAbs were analyzed by SDS-PAGE-immunoblotting essentially as described [10], [12]. Briefly, recombinant proteins separated on SDS-polyacrylamide gels were transferred onto polyvinylidene difluoride (PVDF) membranes (0.45 µm, Millipore, Bedford, MS, USA). The blots were blocked with 1% skimmed milk then incubated with serum samples (1∶5 dilution in Tris-buffered saline, pH 7.5 containing 0.05% Tween 20 [TTBS] and 1% skim milk) at 4°C for 16 h. Alternatively, the blots were incubated with diluted culture supernatants of hybridomas (1∶10 dilution in TTBS and 1% skim milk) for 1 hr at room temperature. After washing, the blots were reacted with phosphatase or peroxidase-conjugated secondary antibodies for 1 h before washing and developing in substrate solutions for antigen-antibody binding visualization and recorded by photography [2], [10], [12]. The intensities of bands on the immunoblots were quantified with the AlphaEaseFCTM software (version 4.0.0, Alpha Innotech Corpration, San Leandro, CA, USA). Each immunoblot analysis was repeated at least three times. The percentage of reduction in immunoreactivity was expressed as the difference between the intensity of WH9 against the wild-type Der p 7 and that against the Der p 7 mutant divided by the intensity of WH9 against the wild-type Der p 7 and then multiplied by 100.

### Immunoblot inhibition

For immunoblot inhibition studies, the culture supernatant from hybridoma WH9 was firstly incubated with 5 µg of purified recombinant wild-type Der p 7 or Der p 7 mutants at room temperature for 2.5 h before incubating with PVDF blots containing purified Der p 7 at room temperature for 1 h. As a control, the same culture supernatant from hybridoma WH9 was pre-incubated with 5 µg of bovine serum albumin (BSA, Pierce, Rockford, Illinois, USA) before immunoblotting against Der p 7.

### Sequencing of heavy chain and light chain variable regions of MoAb WH9

Total RNA from hybridoma WH9 was isolated using a Trizol-reagent (Invitrogen Life Technologies, Inc., Carlsbad, CA, USA) according to the manufacturer's instructions. cDNAs encoding the heavy and the light chains of MoAb WH9 were obtained by reverse transcription with an AffinityScript Multiple Temperature cDNA Synthesis Kit (Stratagene, La Jolla, CA, USA). Sequencing of heavy chain and light chain variable regions were performed with PCR using primers listed in Table 1. The gel purified PCR products were inserted into pGem-T vectors (Promega, Madison, WI, USA) and transformed into M15 competent cells. Random colonies were selected for growth and plasmids extraction. The nucleotide sequences of the cDNA inserts encoded by the isolated plasmids were determined with an ABI 377 automatic sequencer (Applied Biosystems, Foster City, CA, USA). Results were search against Data Bank with the BLAST (http://www. ncbi.nlm.nih.gov/BLAST) program while homologous alignment of sequences were performed with the CLUSTAL14 program. The complementarity determinant regions (CDRs) of WH9 variable domains were defined essentially according to the Kabat system [13].

### Homology modeling of MoAb WH9

Protein homology modeling was performed essentially as described [14]. Firstly, the amino acid sequences of the variable domains of the heavy and light chains of WH9 were compared with entries in the Protein Data Bank for the best matches. The VH of the Fab13b5 fragment in complex with HIV-1 capsid protein (P24) (PDB code: 1e6jH, 81.8% sequence identity) [15] and the VL of the Fab fragment of MoAb MAB 26-2F in complex with human angiogenin (PDB code: 1h0dA, 93.3% identity) [16] were selected as templates for modeling the VH and VL structures of WH9, respectively. In addition, the crystal structure of the orthorhombic form of IgG1 Fab fragment (in complex with an tubulin peptide) with PDB codes of 3qnzB (for heavy chain) and 3qnzA (for light chain) [17], was also selected as the superimposition template. Model building and molecular visualization was carried out using VMD software [18].

### Molecular docking of Der p 7-WH9 complex

Docking of the modeled structure of WH9 and the crystallographic structure of Der p 7 (PDB ID: 3H4Z) [7] was performed using the ZDOCK program [19]. The Der p 7-WH9 complex model was initially subjected to energy minimization using the CHARMM program [20] with harmonic constrained force of 100 (kcal/mol Å^2^) on the backbone atoms, then on the Cα atoms, followed by gradually removing the constraints. A further 5×10^5^ steps of minimization were performed to obtain an optimal Der p 7-WH9 complex structure for further analysis.

## Results

### Determinant of Der p 7 recognized by IgE antibodies

**Figure 1:**
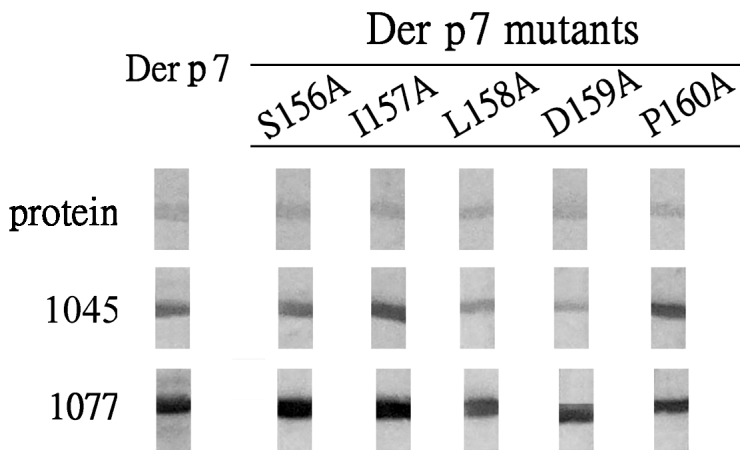
IgE immunoblot activity of serum no. 1045 against Der p 7 and its five point mutants. Serum no. 1077 was included as control. The row labeled as “protein” represents Coomassie blue-stained protein profiles of the wild-type Der p 7 and Der p 7 mutants (S156A, I157A, L158A, D159A and P160A) on PVDF membranes.

In our previous study, Der f 7 peptide Df7-16 (^151^HIGGLSILDPIFGVL^165^) and the corresponding Der p 7 peptide Dp7-16 (^151^HIGGLSILDPIFAVL^165^) inhibited IgE binding to Der f 7 in serum nos. 990 and 1045 of asthmatic patients in a dot-blot inhibition assay [10]. Reduced IgE immunodot blot reactivities against Der f 7 I157A, L158A and D159A mutants were also observed for both serum samples. In addition, D159 contributed to IgE-mediated cross-reactivity between Der f 7 and Der p 7. Furthermore, the wild-type Der f 7 and its mutants (S156A, I157A, L158A, D159A and P160A) have similar far-UV circular dichroism (CD) spectra suggesting these mutations have not changed significantly the overall secondary structure of the proteins [10]. In this study, wild-type Der p 7 and its five mutants have similar CD spectra (data not shown) which are indicative of comparable secondary structures. Among the five Der p 7 mutants prepared (S156A, I157A, L158A, D159A, P160A), serum no. 1045 showed reduced IgE immunoblot reactivity against Der p 7 L158A and D159A mutants (Fig. 1). Serum no. 1077 was included as a control and it reacted with Der p 7 and its five mutants (Fig. 1). Serum no. 1077 has IgE-binding activity against Der f 7 and Der p 7, but peptides Df7-16 and Dp7-16 cannot inhibit its IgE-binding activity (data not shown). Serum no. 862 without IgE antibody against Der p 7 was used as negative control. It showed negative IgE-immunoblot reactivity against Der p 7 and its five mutants (data not shown).

### Determinant of Der p 7 recognized by MoAb WH9

**Figure 2:**
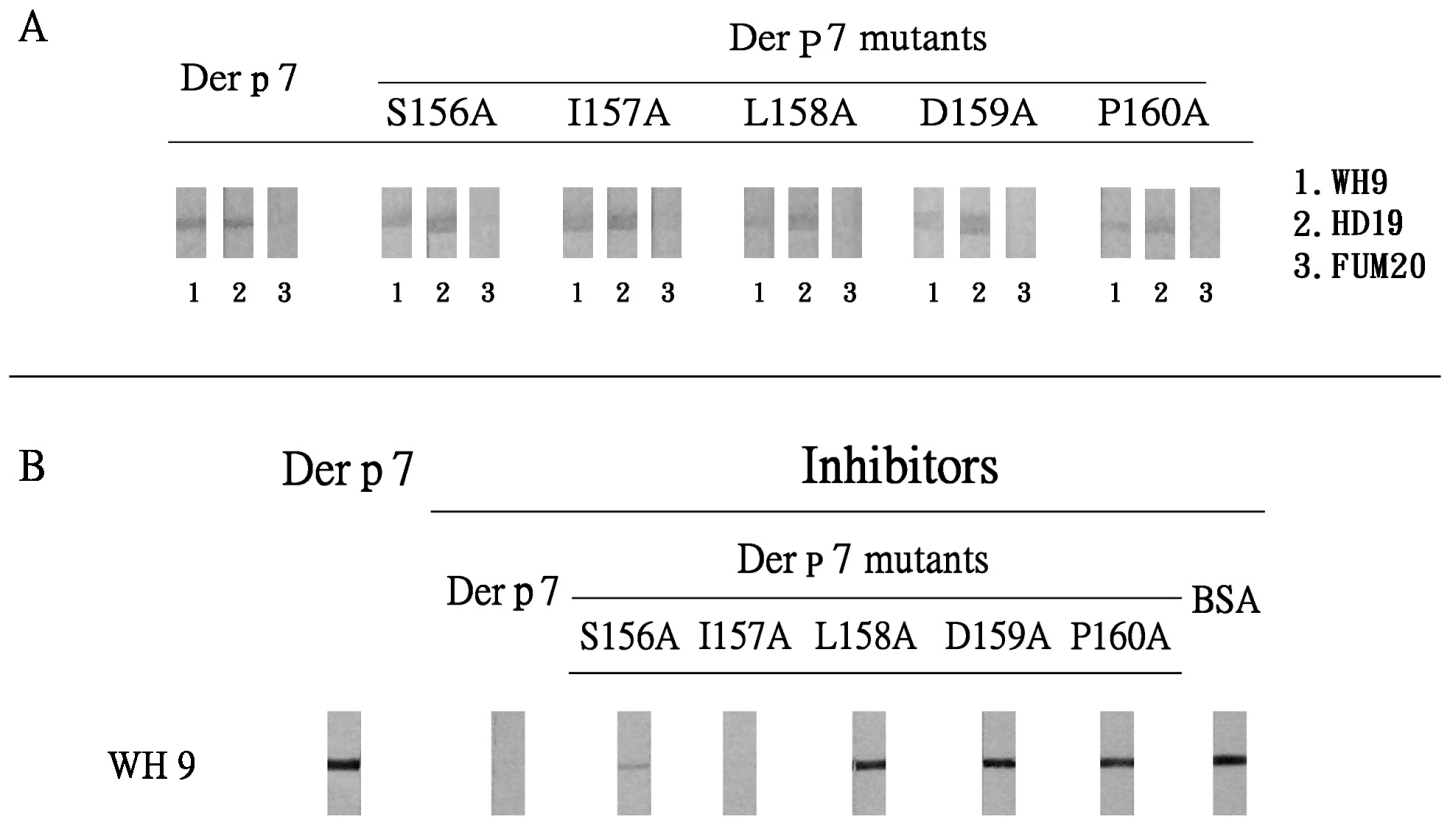
Determinant of Der p 7 recognized by MoAb WH9. (A) Immunoblot activity of MoAb WH9 against Der p 7 and its five mutants. MoAbs HD19 and FUM20 were used as controls. (B) Immunoblot inhibition of WH9-binding against Der p 7 by the wild type and the five Der p 7 point mutants. BSA was included as control.

WH9 reacts with and inhibits IgE-binding against Der p 7 [4]. Thus, WH9 may recognize a determinant on Der p 7 similar to that recognized by IgE antibodies. We show in Fig. 2 (panel A) that WH9 has reduced immunoblot reactivity against Der p 7 L158A, D159A and P160A mutants and to a lesser extent against the Der p 7 S156A mutant. The mean percentages of reduction in immunoreactivity of WH9 against Der p 7 S156A, L158A, D159A and P106A mutants were 18%, 87%, 81% and 87%, respectively. For control, reduced immunoblot reactivity against the five Der p 7 mutants was not detected when MoAb HD19 [4] which recognizes a different determinant on Der p 7 was used (Fig. 2, panel A). In addition, immunoblot activity against Der p 7 and the five Der p 7 mutants was not detected when MoAb FUM20 which reacts against serine protease major fungal allergens [12] was used as a control (Fig. 2, panel A).

To confirm the role of amino acids S156, L158, D159 and P160 in WH9-binding against Der p 7, immunoblot inhibition by Der p 7 mutants was performed. Among these mutants, only the Der p 7 I157A and the wild-type Der p 7 (Fig. 2, panel B) can inhibit significantly WH9-binding against Der p 7. In addition, the Der p 7 S156A mutant inhibits about 65% of the WH9-binding against Der p 7. BSA was included as a negative control for these experiments. Thus, inhibition experiments confirmed that S156, L158, D159 and P160 contribute to WH9-binding against Der p 7. These experiments have been repeated at least three different times and representative results are shown in Fig. 2, panels A and B.

### Amino acid sequences and homology modeling of MoAb WH9

**Figure 3:**
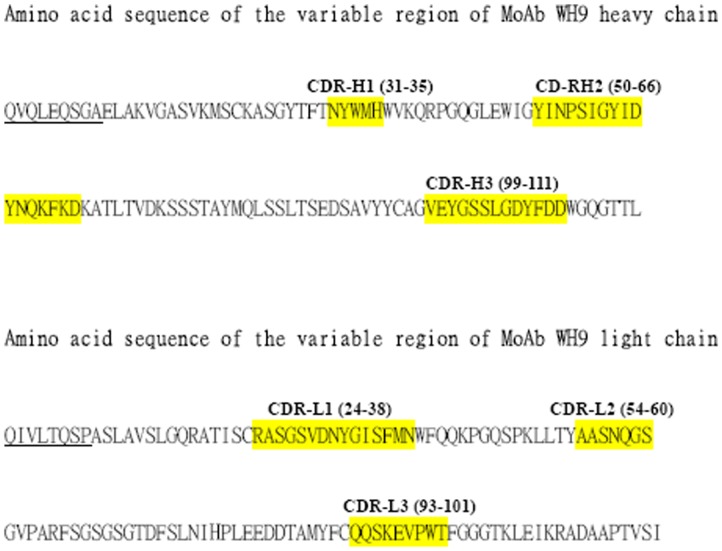
Amino acid sequences of the CDR regions in the variable domains of the heavy (upper panel) and the light (lower panel) chains of MoAb WH9. The first nine amino acids of the WH9 heavy chain and the first eight amino acids of the WH9 light chain are derived from the PCR primers and underlined.

**Figure 4:**
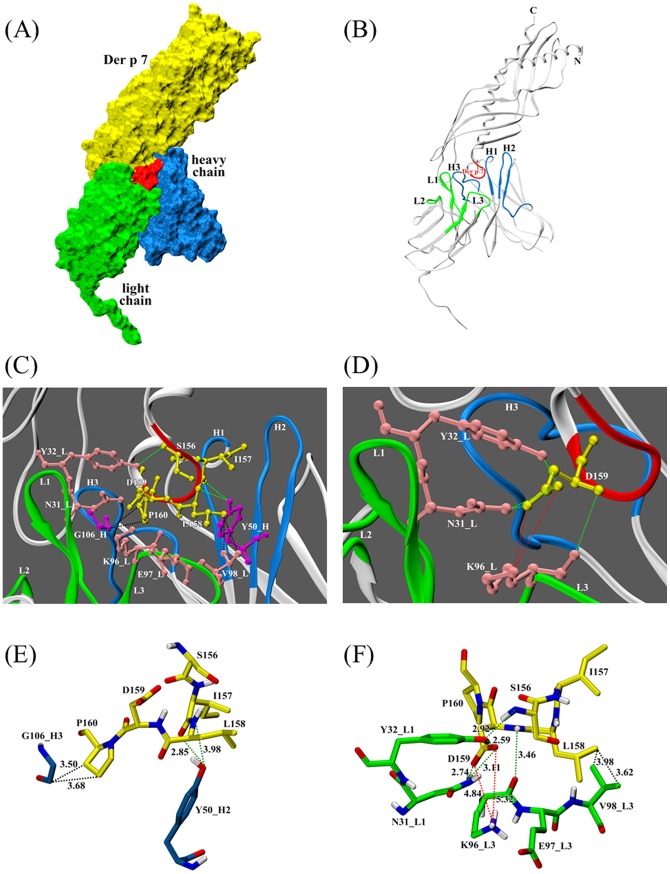
A structural model of the Der p 7-WH9 complex derived from computer-guided modeling and docking. (A) Surface representation of the Der p 7-WH9 complex. The Der p 7 is colored in yellow. The loop-like determinant of Der p 7 (^156^SILDP^160^) recognized by WH9 is colored in red. The variable regions of the heavy chain and the light chain of WH9 are colored in blue and green, respectively. (B) A ribbon presentation of the Der p 7-WH9 complex. The CDRs are colored in blue (VH-CDR) and green (VL-CDR). (C) and (D) Close up view of the potential interface between S156, I157, L158, P160 (in C) and D159 (in D) of Der p 7 and WH9. The potential interacting residues are shown as sticks representation. Potential hydrogen bonds, hydrophobic and electrostatic interactions are indicated in green, black and orange dashed lines, respectively. (E) and (F) Details of potential intermolecular interactions at the Der p 7-WH9 heavy chain (E) and light chain (F) interfaces. Nitrogen and oxygen atoms are shown in blue and red, respectively. Interatomic distances (see Table 2) are depicted and in Å.

The deduced amino acid sequences of the variable domains of the heavy (GenBank accession no. KC222648) and the light (GenBank accession no. KC222649) chains of WH9 were obtained through PCR amplification of WH9 cDNA and summarized in Fig. 3. The first nine amino acids of the WH9 heavy chain and the first eight amino acids of the WH9 light chain were derived from the PCR primers and underlined. The predicted amino acid sequences for the CDRs in the heavy and light chains of MoAb WH9 are highlighted in Fig. 3. The structure of the variable regions of MoAb WH9 was generated with homology modeling and presented in Fig. 4, panels A and B. It has a typical immunoglobulin fold comprising β-sandwich-like structure with two sheets of antiparallel beta strands.

### Structural overview and intermolecular interactions between Der p 7 and WH9

We modeled the binary complex of Der p 7 and WH9 via computational docking. The Der p 7 model in Fig. 4 was adopted from the crystal structure of a Der p 7-MBP complex [7]. Panel A of Fig. 4 shows the two proteins have good shape complementarity and zipped together well. The six CDRs (H1, H2, H3, L1, L2 and L3) on the heavy and light chains of WH9 are essentially loops connecting the β-strands of the variable domains (Fig. 4, panel B). Together they form a cleft for antigen binding. The determinant on Der p 7 also assumes a loop like structure encompassing residues S156, I157, L158, D159 and P160 (Fig. 4, panels B, C and D).

The potential interactions between Der p 7 and WH9 are shown in Fig. 4, panels C and D. The Der p 7-WH9 binary complex model suggests four potential regions that contribute to the binding of Der p 7 with WH9. The loop-like antigenic determinant of Der p 7 (in red) containing residues S156 to P160 is bound into the antigen-binding pocket of WH9 consisting of CDR-H2, CDR-H3 (in blue) as well as CDR-L1 and CDR-L3 (in green). Altogether, five amino acid residues from Der p 7 and six amino acid residues from WH9 form seven hydrogen bonds, four hydrophobic interactions and two electrostatic interactions as summarized in Table 2.

**Table 2:** Summary of the distances between the interaction residues in the modeled Der p 7-WH9 complex.

Chain	MoAb WH9	Der p 7	Distance (Å)
VH			
CDR-H2	OH (Y50)	O (I157)	2.85
	OH (Y50)	N (L158)	3.98
CDR-H3	C (G106)	Cβ (P160)	3.50
		Cγ (P160)	3.68
VL			
CDR-L1	Nδ_2_ (N31)	Oδ_1_ (D159)	2.74
		Oδ_2_ (D159)	3.11
	OH (Y32)	N (S156)	2.92
	OH (Y32)	Oδ_2_ (D159)	2.59
CDR-L3	O (K96)	N (D159)	3.46
	Nζ(K96)	Oδ_1_ (D159)	4.84
		Oδ_2_ (D159)	5.32
	Cγ_1_ (V98)	Cδ (L158)	3.62
	Cγ_2_ (V98)		3.98
	Nζ (K96)…‥Oε_1_ (E97)		4.64
	Nζ (K96)…‥Oε_2_ (E97)		2.61

Of the three CDRs on the heavy chain of WH9, two (CDR-H2 and CDR-H3) are in contact with Der p 7 (Fig. 4, panels C, D, E and Table 2). The side-chain hydroxyl of Y50 of CDR-H2 forms two hydrogen bonds with the main-chain carbonyl oxygen of I157 and the amide nitrogen of L158 on Der p 7. The main-chain carbon of G106 on CDR-H3 makes two hydrophobic interactions with the Cβ and the Cγ of P160 on Der p 7.

Among the three CDRs of the WH9 light chain, residues N31 and Y32 on CDR-L1 as well as K96 and V98 on CDR-L3 are in contact with S156, L158 and D159 of Der p 7 (Fig. 4, panels C, D, F and Table 2). The side chain Nδ_2_ atom of N31 on CDR-L1 makes two hydrogen bonds with Oδ_1_ and Oδ_2_ of D159 on Der p 7. In addition, the side-chain hydroxyl of Y32 of CDR-L1 forms two hydrogen bonds with the amide nitrogen of S156 and Oδ_2_ of D159 on Der p 7. Furthermore, the main-chain carbonyl oxygen of K96 forms a hydrogen bond with the amide nitrogen of D159 on Der p 7. The two Cγ atoms of V98 make two hydrophobic interactions with the Cδ atom of L158 on Der p 7. In addition, the Nζ of K96 forms an electrostatic network among the Oε_1_ and Oε_2_ of E97 on CDR-L3 (Table 2 and Fig. 4, panel F), and the Oδ_1_ and Oδ_2_ of D159 on Der p 7 (Fig. 4, panels D, F and Table 2).

## Discussion

Der p 7 is an important house dust mite allergen associated with human atopic disorders. Using five Der p 7 mutants, we identified L158 and D159 which locate on a loop-like structure are critical amino acid residues contributing to IgE-binding. These two residues are also critical residues contributing to IgE-binding of Der f 7 [10]. In addition to L158 and D159, S156 and P160 also play a crucial role in MoAb WH9-binding against Der p 7. The epitope recognized by WH9 overlaps with the binding sites of IgE against Der p 7 and imply that MoAb WH9 may exhibit IgE-binding inhibition.

We sequenced the amino acid sequences of the variable regions of WH9 (Fig. 3). On alignment, the CDR-H3 region of WH9 has the most variation in length and sequence among mouse MoAbs (data not shown). Through homology modeling and computational docking, we found that four of the six CDRs of WH9 participate in the binding of a loop-like determinant on Der p 7 (Fig. 4). A similar finding has reported for a Hyal epitope composed of nine residues which folded into a helix–turn–helix motive that protrudes away from the protein core and fits into a deep pocket formed by the six CDRs of MoAb 21E11 [21]. In general, loops and areas at the convex or protruding regions of the antigens form the majority of epitopes [10], [22].

In addition to shape complementarity as demonstrated in the modeled Der p 7-WH9 complex (Fig. 4, panel A), our computational docking studies reveal that all five residues (S156, I157, L158, D159 and P160) on the epitope of Der p 7 interact with six residues (Y50 of CDR-H2; G106 of CDR-H3; N31 and Y32 of CDR-L1; and K96 and V98 of CDR-L3) on WH9 (Table 2). It has reported that CDR-H2 together with either the heavy or light chain CDR3 are preferred to play a major role in antigen-antibody interaction [21], [23]–[25]. In this study, the CDR2 of the WH9 light chain does not interact with Der p 7. In the hen egg lysozyme and its mAb D11.15 complex, neither CDR-L1 nor CDR-L2 interacts with the antigen [26]. It is not surprising that Der p 7 does not interact with residues on CDR-L2 which locates in the binding cleft farthest from the antigen (Fig. 4). This observation has also been reported in other antigen-antibody complexes [23].

According to our model, potentially seven hydrogen bonds, two electrostatic and four hydrophobic interactions exist between Der p 7 and WH9 (Fig. 4 and Table 2). Residue L158 on Der p 7 plays an important role in binding to Y50 of CDR-H2 and V98 of CDR-L3 of WH9. It correlates to our findings that the Der p 7 L158A mutant has reduced reactivity with both human IgE and the MoAb WH9 (Figs. 1 and 2). Our results are also in conformity with the conception that aromatic residues, particularly tyrosines, from the Fab portion of the antibody form most of the contacts with an antigen [21], [25], [27].

Our results showed that serum no. 1045 has IgE-binding against Der p 7 but Der p 7 L158A and D159A mutants can reduce this reactivity (Fig. 1). Therefore, residue D159 of Der p 7 is a critical amino acid for IgE-binding. The results are supportive of our recent report that D159 is a critical core residue responsible for an IgE-mediated cross-reactivity between Der f 7 and Der p 7 [10]. Our results also showed that the Der p 7 D159A mutant has reduced reactivity with the MoAb WH9 (Fig. 2) and indicated a role of D159 in WH9-binding against Der p 7. Our modeling experiment suggests that D159 binds MoAb WH9 via four potential hydrogen bonds and two electrostatic interactions (Fig. 4, panel D and Table 2). The wild type Der p 7 and its D159A mutant have similar far-UV circular dichroism spectra (data not shown) and probably comparable secondary structures. Thus, the charged amino acid D159 of Der p 7 plays a pivotal role in binding IgE and MoAb WH9. It is in accordance with those demonstrated previously that charged amino acids play a significant role in allergen-IgE/MoAb interactions [25], [27]–[30]. For example, residue D199 of the Der f 1/Der p 1 allergens is involved in interacting against IgE and the MoAb 4C1 [25]. Similary, residue D68a of a dimerized cockroach allergen Bla g 2 can interact with both the heavy (His-35 of H1) and light (Gln-96 of L3) chains of MoAb 7C11 [27]. Thus, our results are in compliance with reports [9], [21], [25]–[27], [30], [31] that shape complementarity along with hydrogen bonds, electrostatic and hydrophobic interactions contribute to the stability of the antigen-antibody complex.

The Der p 7 S156A and P160A mutants do not reduce the IgE-binding activity in serum no. 1045 (Fig. 1). Residues S156 and P160 are located at the beginning and the end of a loop-like structure on Der p 7 (Fig. 4) and they are amino acid residues contribute to interactions between Der p 7 and WH9 (Fig. 2). Since S156 is located at the fringe of the antigenic loop, the potential hydrogen bond between S156 and Y32 of CDR-L1 possibly has only a minute contribution in stabilizing the Der p 7 and WH9 interaction. Proline is conformationally rigid and locates commonly in turns or at the end of an alpha helix. The P50 of Bet v 1 has observed to form a hydrogen bond with the Y96 on CDR-L3 of MoAb BV16 [31]. In addition, the discrepancy observed between antigenic determinants of an allergen recognized by human IgE and mouse IgG antibodies has also been reported previously [32].

According to our molecular docking experiment, I157 possibly interacts with WH9 but reduced WH9 binding against the Der p 7 I157A mutant has not been detected. I157 may interact with Y50 of CDR-H2 via its carbonyl oxygen. Probably, the replacement of I157 with alanine does not change the local backbone conformation to affect the experimental antigen-antibody binding. Nevertheless, we have recently identified that in addition to the Der f 7 L158A and D159A mutants, the recombinant Der f 7 I157A mutant has reduced IgE-binding activity and contributes to IgE-binding against Der f 7 [10].

We have validated our model by measuring the contact area between the antibody and the epitope. Our Der p 7-WH9 complex has a contact area of 1292.9 Å^2^ (data not shown) and is similar to that of Ig-based antibodies and protein antigens (1400–2300 Å^2^) [33]. It is larger than that of the Der p 1-4C1 complex (750 Å^2^) [25]. The larger contact area suggests stronger nonbonding interactions between the antibodies and antigens. The van der Waals and electrostatic interactions in the Der p 7-WH9 complex are −99.57 and −264.00 kcal/mol, respectively (data not shown), and stronger than those in the Der p 1-4C1 complex (−70.53 kcal/mol for van der Waals interaction and −196.58 kcal/mol for electrostatic interaction). Our results from molecular dynamics (MD) simulation also support the structural stability of the Der p 7-WH9 complex. The root mean square deviations (RMSD), contact area and nonbonding interactions as functions of simulation time all indicate that the predicted structure of Der p 7-WH9 complex is stable and reliable (data not shown).

In summary, we characterized the antigenic determinants of Der p 7 through in vitro mutagenesis experiments and delineated its interactions with an IgE-binding inhibition MoAb WH9 through in silico antibody structure modeling and computational docking. In the absence of available X-ray deflection models, our study provides an alternative and powerful approach to characterize allergenic epitopes and demonstrates possible interactions between allergens and their specific antibodies at molecular level. Allergen-specific, IgE-binding-inhibition antibodies have been considered and tested in specific passive immunotherapy [34], [35]. The analysis model system we established and results obtained from the present study may provide important bases which will facilitate therapeutic developments of active hypoallergenic vaccines as well as passive blocking antibodies against human atopic diseases.
